# The role of harsh parenting practices in early‐ to middle‐childhood socioemotional development: An examination in the Millennium Cohort Study

**DOI:** 10.1111/cdev.13761

**Published:** 2022-03-25

**Authors:** Lydia Gabriela Speyer, Yuzhan Hang, Hildigunnur Anna Hall, Aja Louise Murray

**Affiliations:** ^1^ Department of Psychology University of Edinburgh Edinburgh UK; ^2^ Department of Psychology University of Cambridge Cambridge UK

## Abstract

Patterson's coercion model describes a gradual escalation in maladaptive parent–child transactions whereby externalizing behaviors lead to increases in maladaptive parenting and vice versa. The current study investigates the role of (predominantly mother‐reported) harsh parenting practices in the within‐person development of conduct problems, hyperactive/inattentive behaviors, and emotional problems. A random‐intercept cross‐lagged panel model was fit across ages 3, 5, and 7 (*N* = 14,037, 49% female, 84% White) using the UK population‐representative Millennium Cohort Study. Findings support Patterson's coercion model, providing evidence for reciprocal within‐family relations between parenting practices and child behaviors. They suggest the importance of addressing parenting difficulties in families where children present with socioemotional difficulties in order to help prevent the accumulation of additional issues.

**Abbreviations**
ADHD: attention deficit hyperactivity disorder
BIC: Bayesian information criterion
CFI: comparative fit index
CLPM: cross‐lagged panel model
CTS: Conflict Tactics Scale
MCS: Millennium Cohort Study
MLR: robust maximum likelihood estimator
OSF: Open Science Framework
RI‐CLPM: random‐intercept cross‐lagged panel model
RMSEA: root mean squared error of approximation
SDQ: Strengths and Difficulties Questionnaire
TLI: Tucker Lewis index

Socioemotional difficulties in childhood are one of the best predictors of adverse developmental outcomes, including later diagnoses of mental health disorders, academic‐underachievement, and delinquency (e.g., Hammer et al., 2017). Among the most prevalent childhood socioemotional problems are conduct problems, emotional problems, and hyperactive and inattentive behaviors (Danielson et al., 2018; Ghandour et al., 2019). These issues often co‐occur (Kessler et al., 2005) and when they do, this further increases the likelihood of negative developmental outcomes (Sibley et al., 2011). Understanding risk factors in the development of socioemotional problems early in life is therefore important for informing interventions that can improve the likelihood of children following healthy developmental trajectories.

One factor that has consistently been linked to increased socioemotional difficulties, especially externalizing behaviors, is maladaptive parenting (Pinquart, 2017a). Harsh parenting techniques, which include both verbal (e.g., shouting) and physical (e.g., spanking) punishment, are still fairly common, particularly up until middle childhood (Lansford et al., 2009). However, a large number of studies have found that such parenting techniques negatively affect children's socioemotional development (Bauer et al., 2021; Pinquart, 2017a, 2017b). In response, policies to reduce harsh parenting have recently been implemented in some parts of the United Kingdom. In Scotland, physical punishment of children was prohibited in 2021 with a similar ban also coming into force in Wales in 2022 (Justice Directorate, 2020). In England and Northern Ireland, however, physical punishment of children is still officially permitted as long as it is a “reasonable punishment” that does not lead to more than a “transient or trifling” injury (Crown Prosecution Service, 2020).

Considering that children with behavioral problems can be expected to elicit different parenting strategies, a number of studies have investigated whether the observed associations between harsh parenting and externalizing problems in children are in fact driven by harsher parenting being employed in an attempt to handle children with more difficult temperaments (Yan et al., 2021). Indeed, models such as Patterson's coercion model view the association between parenting and behavioral problems as bidirectional (Patterson, 2002). Specifically, Patterson's model suggests that children with behavioral problems elicit maladaptive parenting from their caregivers which in turn escalate rather than reduce their child's externalizing behaviors.

Overall, however, evidence for Patterson's coercion model has been mixed (Besemer et al., 2016; Lansford et al., 2012; Rolon‐Arroyo et al., 2018; Yan et al., 2021). For instance, a study by Lansford et al. (2012) using a sample of 477 European American families found that externalizing behaviors at age 5 predicted increases in mother‐reported spanking, yelling, and denial of privileges at ages 6 to 8 while spanking predicted increases in externalizing behaviors. In contrast, Besemer et al. (2016) did not identify any significant effects of maladaptive parenting behaviors on externalizing problems or vice versa in a sample of 503 parent‐son dyads aged 6 to 13.

One of the potential reasons for the mixed findings on this may be related to the fact that most studies have not primarily focused on the within‐family effects that are implied by Patterson's model, but on rank‐order changes. Such changes are considerably influenced by relatively stable between‐family differences such as genetic predispositions or family adversity and consequently confound estimates of within‐family relations (Lansford et al., 2012; Rajyaguru et al., 2019). This is problematic considering that interventions aiming to interrupt the coercive cycle posited by Patterson would have to be aimed at processes occurring at the within‐family level. The need for appropriate statistical techniques that can operationalize the processes implied by developmental psychopathology models has resulted in the advancement of several methods that are suited to disaggregating within‐ from between‐person effects (Hamaker et al., 2015; Speyer et al., 2020). In particular, extensions to the cross‐lagged panel model (CLPM) such as the Random‐Intercept CLPM (RI‐CLPM; Hamaker et al., 2015) or the autoregressive latent trajectory model with structured residuals now offer accessible tools to appropriately operationalize the within‐family processes implied by developmental models such as Patterson's coercion model.

One of the few studies to date that has tested Patterson's model using a statistical operationalization that achieves the necessary disaggregation of between‐ and within‐family effects was conducted by Besemer et al. (2016). They investigated the bidirectional relations between different parenting behaviors (physical punishment, parental involvement, parent–child communication) and several types of externalizing problems (interpersonal callousness, conduct and oppositional defiant problems, hyperactivity/impulsivity) in a sample of 503 parent‐son dyads across eight time points (ranging in ages from 6 to 13 in 6‐month intervals). In contrast to much of the previous literature, they did not identify any significant effects of maladaptive parenting behaviors on externalizing problems or vice versa, suggesting that these relations might not actually play out within families but reflect between family differences (Besemer et al., 2016). Building on Besemer et al.’s work, Rolon‐Arroyo et al. (2018) investigated reciprocal relations between certain parenting behaviors and conduct disorder symptoms in a sample of 199 preschoolers aged 3 to 6 that were selected based on them exhibiting high levels of conduct problems. When disaggregating within‐ and between‐person effects, they found some evidence for maternal over‐reactivity and decreased maternal warmth predicting increases in conduct disorder symptoms. In addition, conduct disorder symptoms predicted increases in paternal over‐reactivity. They did not, however, identify any child‐to‐mother or father‐to‐child effects (Rolon‐Arroyo et al., 2018). The generalizability of these studies may, however, be limited. In particular, Rolon‐Arroyo et al. (2018) used a sample selected for and thus potentially range‐restricted on conduct problems, while Besemer et al. (2016) only investigated bidirectional relations between parenting and externalizing problems in parent‐son dyads in school‐aged children. It is important to extend this work to earlier ages as the hypothesized reciprocal effects may be more pronounced when behavioral problems first emerge and when harsh parenting behaviors are most widely used, that is in early‐ to middle‐childhood.

Early‐ to middle‐childhood is a particularly important period for studying the development of socioemotional difficulties as this is the age at which many of these difficulties may begin to emerge. In addition, the preschool years have also been suggested to be one of the most central periods for parent‐child interactions in the context of coercive family processes (Smith et al., 2014). Early‐ to middle‐childhood is also the developmental period in which harsh‐parenting behaviors have been found to be most widely used. Prior research has indicated that parents start to decrease their use of such parenting behaviors when children can be more easily reasoned with and have more volitional control over their behaviors, that is during middle childhood (Giles‐Sims et al., 1995; Lansford et al., 2009). During the preschool years, children typically acquire a range of important executive function and self‐regulation skills that, for instance, allow them to inhibit inappropriate behavioral responses (Montroy et al., 2016).

However, within the early‐to‐middle childhood period, important developmental changes occur that may have implications for the processes implied by models such as Paterson's coercive cycles. Accompanying increases in self‐regulation ability (Montroy et al., 2016), the levels and manifestation of different behavior problems evolve during this period (Matthys & Lochman, 2016). For example, physical aggression overall decreases between toddlerhood and school age, while indirect aggression shows a relative increase (Côté et al., 2007). Similarly, while compliance overall increases over this period, it also shifts in form from less overt manifestations (ignoring a parent or throwing a tantrum) to more subtle and sophisticated forms such as attempting to negotiate (Kuczynski & Kochanska, 1990).

The transition to school also represents an important transition with respect to reciprocal parent‐child models. At this stage, teachers become an important additional adult influence on children. While they may be an opposing force to negative parental influences, it has also been proposed that they can enter similar reciprocal cycles which could reinforce children's difficulties (Sameroff & Mackenzie, 2003). Thus, while it is known that there are substantial developmental changes in children's behavior and social environments in early‐to‐middle childhood, the extent to which reciprocal relations between child behavior and parenting practices are affected by these changes is not well understood.

In addition, little is so far known on whether these effects differ by child sex. It is well established that there are sex differences in children's socioemotional development with boys being proportionately more likely to exhibit conduct problems compared to girls whereas girls are more likely experience emotional problems such as anxiety and depression (Lewis et al., 2020; Moffitt et al., 2001). Boys are also more than 3 times as likely to be diagnosed with attention deficit hyperactivity disorder (ADHD), however, diagnostic criteria are likely male‐biased in that girls show different symptom patterns and later onsets that are less in line with current diagnostic criteria (Murray et al., 2019). Given that Patterson's coercion model is primarily focused on conduct problems, much of the research to date has focused on boys rather than girls (e.g., Besemer et al., 2016) with little research exploring sex differences. One of the few studies to date that did also consider sex differences, however, found no significant sex differences in the relations between parenting behaviors and conduct problems (Rolon‐Arroyo et al., 2018). Considering these limitations and the overall inconclusive evidence for Patterson's coercion model, it is important to investigate the bidirectional within‐family relations of harsh parenting practices and child behavior from early in life using large, population representative samples.

Research also points to the potential importance of internalizing problems such as anxiety and depression as an outcome of the coercive cycle of parent–child interaction proposed by Patterson, as these commonly co‐occur with behavioral problems (Gnanavel et al., 2019; Kessler et al., 2005). A number of studies have linked maladaptive parenting to internalizing problems (Pinquart, 2017b). Following Capaldi’s (1992) dual failure model which hypothesizes directional relations from externalizing problems to internalizing problems via problems in the social and academic domains, some studies have investigated whether parenting practices mediate the relation between behavioral problems and internalizing problems. These studies have found evidence for maternal dissatisfaction mediating developmental cascades from externalizing problems to internalizing problems (Wertz et al., 2015) and for ineffective behavioral management mediating cascades from ADHD symptoms to depression (Ostrander & Herman, 2006). However, evidence has not been consistent, with some studies finding no evidence for mediation effects (Gair et al., 2021). On the whole, parenting behaviors have only rarely been investigated as a potential mediator in within‐person relations between externalizing and internalizing problems as studies investigating such links have predominantly focused on the mediators originally hypothesized by the dual failure model, that is, peer problems and academic achievement (Capaldi, 1992). Considering that there has been ample evidence for influences of parenting behaviors on both internalizing and externalizing problems (Pinquart, 2017a, 2017b), it would be valuable to investigate such processes within a more synthetic framework that unites aspects of both the dual failure model and Patterson's coercion model.

While the effect of harsh parenting on child development has been relatively widely researched, less is known about other disciplinary parenting practices, such as withdrawal tactics. Parenting tactics such as sending the child to their bedroom as reactions to negative behaviors have sometimes been viewed as positive parenting techniques (Morawska & Sanders, 2011; Rajyaguru et al., 2019), however, some evidence has also indicated that such tactics may be associated with increased socioemotional difficulties (Gershoff et al., 2010; Lansford et al., 2012). Considering the limited and so far inconclusive evidence on the effect of withdrawal tactics on child development, further exploratory research that can help pave the way for more confirmatory studies is needed.

In the current study, we investigate the role of (predominantly mother‐reported) harsh parenting tactics (e.g., smacking, shouting), in the development of socioemotional difficulties across early‐ to middle‐childhood as this is the period in which harsh parenting behaviors are most commonly used and at which socioemotional problems tend to emerge. Specifically, we investigate whether these parenting practices show bidirectional relations with two different externalizing behaviors (conduct problems and hyperactive/inattentive behaviors) as well as emotional problems. To analyze these within‐family relations, we fit a RI‐CLPM across ages 3, 5, and 7 using data (*N* = 14,037) from the UK population representative Millennium Cohort Study (MCS). Considering that early‐ to middle‐childhood is a period in which many developmental changes occur that may have implications for the processes implied by Patterson's coercion model, we allowed for time‐varying effects from age 3 to age 5 as well as from age 5 to age 7 in our model as it may be possible that the effects of harsh parenting vary by age. We hypothesized that, in line with Patterson's coercive model of parenting (Patterson, 2002), harsh parenting tactics share reciprocal positive relations with conduct problems and hyperactive/inattentive behaviors. We did not have any specific hypotheses on differential effects at different stages of development as it is not a‐priori clear what these effects may be. In addition to testing Patterson's coercion model, we aimed to test the plausibility of a more integrated developmental cascade model which unites Patterson's coercion model with the dual failure model by investigating whether parenting tactics mediate links between behavioral and emotional problems. We hypothesized that harsh parenting tactics mediate developmental cascades from conduct problems and hyperactive/inattentive behaviors to increased emotional problems. While harsh parenting tactics have often been the focus of studies investigating the relations between parenting practices and socioemotional difficulties (Bauer et al., 2021), withdrawal tactics (e.g., taking away treats, ignoring) have only rarely been included in such studies even though there has been some evidence for such tactics also leading to increased parent–child conflict (Gershoff et al., 2010; Lansford et al., 2012). In addition to investigating whether harsh parenting shows bidirectional relations with children's socioemotional difficulties, we therefore further investigate the relations between (predominantly mother‐reported) withdrawal tactics and children's socioemotional development taking an exploratory approach.

## METHODS

### Participants

Participants of the current study were families taking part in the MCS (Connelly & Platt, 2014; Joshi & Fitzsimons, 2016) The MCS is a longitudinal birth cohort study based in the United Kingdom that has been following the lives of around 19,000 children and their families from shortly after birth up until age 17 with data collection still ongoing. Children born between September 2000 and January 2002 were sampled from all four UK nations using a stratified sampling procedure clustered by electoral wards. The MCS further intentionally oversampled regions of high ethnic minority density and poverty in order to ensure that the sample is representative of the UK population. Eligible children were selected based Child Benefit records from the British Government Department of Work and Pensions. The first wave of data collection took place when children were 9 months old with subsequent waves taking place at ages 3, 5, 7, 11, 14, and 17. As most birth cohort studies, the MCS is subject to attrition. At the age 3 wave, 81% of the eligible sample participated (*N* = 15,590). This reduced to 79.2% (*N* = 15,246) at the age 5 wave and 72% at the age 7 wave (*N*=13,857). Since eligible families continued to be invited to participate in future waves of the MCS (unless they indicated they wanted to permanently withdraw from the study), some of the families that did not participate at the age 5 wave rejoined the study at the age 7 wave. To account for non‐random dropout and its complex sampling design, the MCS provides attrition weights as well as stratification and clustering variables that should be incorporated into analyses to correct the sample to be representative of the population. For further details, see the MCS cohort profiles (Connelly & Platt, 2014; Joshi & Fitzsimons, 2016). The current study included all children who were participating up to the age 7 wave (*N* = 14,037). For demographic characteristics (see Table 1).

**TABLE 1:** Demographic characteristics

Variable	Category	%	*N*
Sex	Female	49.36	6929
	Male	50.64	7108
Child ethnicity	White	84.42	11,407
	Other ethnicity	15.58	2105
Maternal academic qualification	Higher degree	3.65	492
	First degree	14.35	1937
	Diplomas in higher education	9.07	1224
	A/AS/S levels	9.91	1337
	O level/GCSE grades A–C	33.78	4559
	GCSE grades A–C	10.04	1355
	Other academic qualification	2.50	338
	None of these qualifications	16.70	2253
Deprivation	Most deprived decile	13.98	1793
	10%–<20%	12.62	1619
	20%–<30%	11.42	1464
	30%–<40%	10.03	1286
	40%–<50%	9.44	1211
	50%–<60%	8.76	1123
	60%–<70%	7.65	981
	70%–<80%	7.90	1013
	80%–<90%	8.90	1141
	Least deprived decile	9.31	1194
		***M***	***SD***
Age	Wave 2	3.13	.20
	Wave 3	5.22	.25
	Wave 4	7.23	.25

GCSE, general certificate of secondary education.

### Ethical considerations

The MCS was approved by the London Multicentre Research Ethics Committee and is funded by the UK Economic and Social Research Council (Shepherd & Gilbert, 2019). Consent was obtained from all participating parents at each sweep.

### Procedure

In the MCS, trained interviewers visited cohort members’ homes and collected data through a combination of face‐to‐face interviews and self‐complete questionnaires. At waves 2, 3, and 4 (i.e., when children were median‐aged 3, 5, and 7), self‐completion questionnaires included the Strengths and Difficulties Questionnaire (SDQ) and the Straus Conflict Tactics Scale (CTS). These were completed by the primary caregiver (98% mothers).

### Measures

Socioemotional problems were measured using the parent‐reported version of the SDQ (Goodman, 1997) The SDQ is a behavioral screening tool assessing children's psychosocial development across five domains: emotional problems, peer problems, conduct problems, hyperactivity/inattention, and prosocial behavior. For each subscale, parents were asked to rate their child's behavior on five items that are scored on a three‐point Likert scale (“not true,” “somewhat true,” “certainly true”). These item scores were summed up to create a subscale score (range: 0–10) with lower scores indicating fewer socioemotional problems, except for the prosocial score where lower scores indicate less prosocial behavior. In the MCS, SDQs were predominantly completed by the children's mothers (~98%). For the age 3 wave in the MCS, the SDQ was adapted for age appropriateness, modifying two items in the conduct problems subscale and one item in the hyperactivity/inattention subscale. In the current study, the subscale scores for conduct problems, emotional problems, and hyperactivity/inattention were used as measures of socioemotional functioning. The SDQ has been found to generally have good psychometric properties (for a review, see Kersten et al., 2016) and shows invariance across development (ages 5–14), sex, and informants in the MCS (Murray et al., 2020, 2021).

Disciplinary parenting practices were assessed using six items from the CTS (Straus, 2019). The CTS was developed to measure negative parental conflict tactics such as physical and emotional violence toward their child in the past year. Psychometric analyses have found support for discriminant and construct validity (Straus et al., 1998). In the MCS, primary caregivers (primarily mothers, ~98%) were asked about how they often they used harsh parenting tactics (shouting, smacking, telling off) or withdrawal tactics (ignoring, sending to bedroom/naughty chair, taking away treats) when their child did not behave well. Items were scored on a five‐point Likert scale (“never,” “rarely,” “once a month,” “at least once a week,” “daily”). Individual items from each discipline category were summed up to create two continuous parenting scores for harsh parenting tactics and withdrawal tactics. Descriptive statistics for SDQ subscale scores and parenting scores, including measures of internal consistency, are presented in Table 2.

**TABLE 2:** Descriptive statistics

	*N*	*M*	*SD*	Min	Max	Skew	Kurtosis	Omega
Age 3 emotional problems	12,194	1.363	1.490	0	10	1.529	3.056	.78
Age 5 emotional problems	13,005	1.386	1.597	0	10	1.514	2.638	.77
Age 7 emotional problems	13,626	1.540	1.776	0	10	1.449	2.149	.79
Age 3 hyperactivity/inattention	12,102	3.888	2.362	0	10	0.446	−0.356	.68
Age 5 hyperactivity/inattention	12,955	3.281	2.375	0	10	0.680	−0.027	.77
Age 7 hyperactivity/inattention	13,605	3.375	2.526	0	10	0.648	−0.242	.80
Age 3 conduct problems	12,217	2.798	2.054	0	10	0.735	0.268	.74
Age 5 conduct problems	13,023	1.503	1.504	0	10	1.175	1.577	.75
Age 7 conduct problems	13,655	1.398	1.553	0	10	1.387	2.300	.79
Age 3 withdrawal tactics	10,418	5.176	2.816	0	12	0.212	−0.709	.62
Age 5 withdrawal tactics	12,420	5.087	2.185	0	12	0.050	−0.216	.66
Age 7 withdrawal tactics	13,042	4.697	2.149	0	12	0.116	−0.133	.70
Age 3 harsh parenting tactics	10,703	6.381	2.377	0	12	−0.113	−0.458	.76
Age 5 harsh parenting tactics	12,527	5.385	1.952	0	12	0.086	−0.183	.75
Age 7 harsh parenting tactics	13,193	5.099	1.915	0	12	0.130	−0.220	.76

Note

Omega calculated using polychoric item correlations to account for ordinal responses.

### Statistical analysis

In order to appropriately operationalize the within‐family processes implied by Patterson's model of coercive parenting, a RI‐CLPM was fitted. The RI‐CLPM disaggregates within‐ from between‐person effects by including random intercepts for each repeatedly measured variable. These are then allowed to covary. Cross‐lagged and autoregressive effects are defined between the residuals (reflecting deviations from the person/family‐specific means), giving insights into within‐family processes (Hamaker et al., 2015). For a schematic illustration of a two‐outcome RI‐CLPM (see Figure S1). In addition to all first‐order autoregressive and cross‐lagged effects, within‐time residual covariances were estimated in the model. In order to test for longitudinal mediation from hyperactive/inattentive behaviors to conduct problems and emotional problems via (predominantly mother‐reported) parenting practices as well as from conduct problems to emotional problems via parenting practices, second‐order cross‐lagged effects were included from hyperactive/inattentive behaviors and conduct problems at age 3 to emotional to emotional problems at age 7. Given established sex differences in socioemotional development (Lewis et al., 2020; Moffitt et al., 2001; Murray et al., 2019) we first tested a multigroup model in which all autoregressive and cross‐lagged parameters were constrained to be equal across boys and girls. We then compared this model to a model in which only the constraints necessary for identification were imposed. To evaluate whether the unconstrained model fit better than the constrained model, we compared Bayesian information criterion (BIC) estimates. Since these results suggested that the BIC was better for the constrained model (∆BIC = 421.679), we proceeded with a combined model for girls and boys but additionally regressed the intercept factors on sex in order to adjust for sex differences in baseline levels of (predominantly mother‐reported) parenting behaviors and socioemotional difficulties. Full results of constraint and unconstraint multigroup models are available on the Open Science Framework (OSF): https://osf.io/7g9dk/.

The RI‐CLPM was fitted in *Mplus 8*.*5* (Muthén & Muthén, 2018) using a robust maximum likelihood estimator (MLR), implying a full information maximum likelihood approach to addressing missing data. When using MLR, standard errors are calculated using the delta method which has been found to be too conservative for assessing the significance of mediation effects (MacKinnon et al., 1995). As such, we also estimated bootstrapped 95% confidence intervals with standard maximum likelihood estimation. Stratification and clustering variables as well as attrition weights were included in all models to account for the complex sampling design of the MCS and non‐random dropout. Model fit was judged to be acceptable if the root mean squared error of approximation (RMSEA) was <.05 and comparative fit index (CFI) and Tucker Lewis index (TLI) were >.90. Full model results and corresponding code are available on the OSF: https://osf.io/7g9dk/.

Finally, given that the RI‐CLPM is vulnerable to unmeasured confounding by time‐varying influences at the within‐person level for our mediation analyses, we conducted a sensitivity analysis for our mediation analyses. In particular, we calculated “failsafe” statistics to estimate how large the unmeasured confounding would have to be to attenuate our mediation effects to null (Kenny, 2013). For unmeasured confounding to occur, the unmeasured influences need to be related both to the mediator and outcome, therefore, the following formula can be used to estimate a failsafe “*ef*” where *e* is the path from unmeasured confounders to the mediator, and *f* is the path from the unmeasured confounders to the outcome:

(1) $$\mathrm{Standardized}\;ef=\frac{r_{MY.X}S_{M.X}S_{Y.X}}{S_{M}S_{Y}},$$

where $r_{MY.X}$ is the correlation between *M* and *Y* controlling for *X*, $S_{M.X}$ is the standard deviation of *M* after the variance due to *X* is removed, $S_{Y.X}$ is the standard deviation of *Y* with the variance due to *X* removed and $S_{M}$ and $S_{Y}$ are the standard deviations of *M* and *Y*. The logic of failsafe *ef* is that if the magnitude of *e* and *f* are implausibly large then it is unlikely that the observed mediating effect is entirely driven by unmeasured confounders. Values for *e* and *f* can be estimated by assuming *e* = *f* and therefore taking the square root of the failsafe *ef* derived using Equation (1) above.

## RESULTS

**FIGURE 1:**
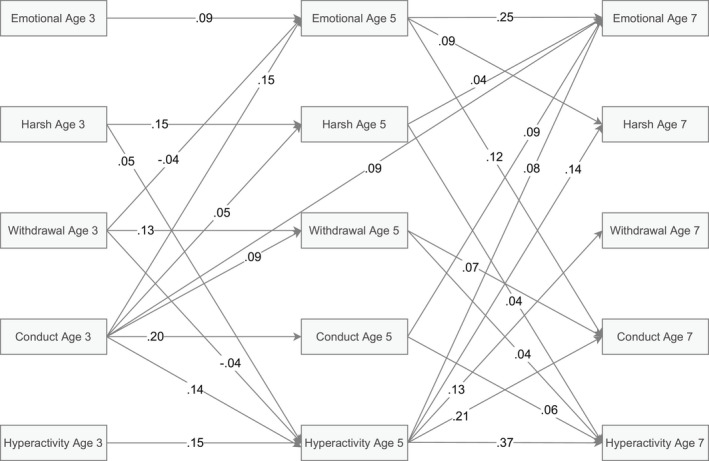
Standardized autoregressive and cross‐lagged parameters. Emotional = emotional problems, Harsh = harsh parenting tactics, Withdrawal = withdrawal tactics, Conduct = conduct problems, Hyperactivity = hyperactive/inattentive behaviors. Only statistically significant paths are shown. Random intercepts and covariance parameters are omitted for clarity

The RI‐CLPM showed excellent fit according to RMSEA = .023, CFI = .997 and TLI = .981. Standardized autoregressive and cross‐lagged parameters are summarized in Figure 1 and presented in full in Table S1. Emotional problems and hyperactive/inattentive behaviors were stable across both lags (age 3–5 and age 5–7) whereas conduct problems and both forms of (predominantly mother‐reported) disciplinary parenting practices were stable only across the first lag. In terms of parent‐to‐child effects, harsh parenting tactics were associated with more hyperactive/inattentive behaviors across both lags and, at age 5, with more emotional problems at age 7. Withdrawal tactics at age 3 were associated with fewer hyperactive/inattentive behaviors and emotional problems at age 5, however, this direction was reversed from age 5 to 7 where withdrawal tactics were associated with more hyperactive/inattentive behaviors and conduct problems. With regards to child‐to‐parent effects, conduct problems at age 3 were associated with increased harsh parenting and withdrawal tactics at age 5, whereas hyperactive/inattentive behaviors at age 5 were associated with increased harsh parenting and withdrawal tactics at age 7. In addition, emotional problems at age 5 were also associated with increased harsh parenting at age 7. For residual correlations between all constructs at each wave, see Table 3. These indicate that all constructs showed moderate to strong concurrent within‐family associations.

**TABLE 3:** Residual correlations

	Age 3					Age 5					Age 7				
	1.	2.	3.	4.	5.	1.	2.	3.	4.	5.	1.	2.	3.	4.	5.
1. Emotional problems	—					—					—				
2. Hyperactivity/inattention	.178*	—				.220*	—				.229*	—			
3. Conduct problems	.263*	.382*	—			.283*	.407*	—			.345*	.399*	—		
4. Withdrawal tactics	.020	.083*	.228*	—		.095*	.189*	.234*	—		.142*	.251*	.325*	—	
5. Harsh parenting tactics	.072*	.181*	.298*	.346*	—	.154*	.188*	.191*	.298*	—	.140*	.238*	.255*	.364*	—

* *p* < .05.

Evaluating whether (predominantly mother‐reported) disciplinary parenting practices mediated cascades from hyperactive/inattentive behaviors and conduct problems to emotional problems, no evidence for effects of hyperactive/inattentive behaviors on emotional problems via parenting was found. However, results indicated that the combined effect of harsh parenting and withdrawal tactics mediated a cascade from conduct problems at age 3 to emotional problems at age 7, while neither harsh parenting nor withdrawal tactics were significant mediators on their own. Bootstrapped analyses further confirmed these findings and additionally suggested that harsh parenting tactics may have a small mediating effect in cascades from conduct problems to emotional problems. For parameter estimates of the MLR analysis as well as bootstrapped confidence intervals of indirect effects, see Table 4.

**TABLE 4:** Indirect effects

	*β*	*SE*	*p*	CIlower	CIupper	Failsafe ef
Age 3 conduct problems to age 7 emotional problems						
Via age 5 harsh parenting tactics	.002	.001	.140	.00001	.005	.190
Via age 5 withdrawal tactics	.003	.002	.131	−.001	.007	.170
Sum of indirect effect	.004	.002	.041	.001	.010	
Age 3 hyperactivity/inattention to age 7 emotional problems						
Via age 5 harsh parenting tactics	.000	.001	.700	−.002	.002	.190
Via age 5 withdrawal tactics	.000	.001	.584	−.001	.002	.170
Sum of indirect effect	.001	.001	.584	−.002	.004	

Note

Confidence intervals (CIs) are based on 1000 bootstrapped samples using standard maximum likelihood estimation.

Failsafe *ef* was calculated from the fully standardized solutions for each mediating effect as a sensitivity analysis. The full calculations are available in Appendix S1 and Table S2. The failsafe *e* and *f* for the path from age 3 conduct problems to age 7 emotional problems via age 5 harsh parenting was *β* = .19. That is, the paths from an omitted confounder to the mediator and outcome would both need to be as large as *β* = .19. In the context of within‐person effects in RI‐CLPMs this is a reasonably large effect (e.g., similar in magnitude to the largest cross‐lagged effect we found in our model overall, and similar in magnitude to other studies using RI‐CLPMs, e.g., Oh et al., 2020). As such, it may be unlikely that this entire indirect effect is attributable to unmeasured within‐person confounding. Of note, the relatively small effect sizes observed here may still represent important effects given the longitudinal modeling context, see elsewhere for details (Adachi & Willoughby, 2015).

## DISCUSSION

The aim of the current study was to investigate the role of (predominantly mother‐reported) harsh parenting practices in the development of socioemotional problems in early‐ to middle‐childhood. Following Patterson's coercion model, we hypothesized that conduct problems and hyperactive/inattentive behaviors would share reciprocal relations with harsh parenting tactics. This hypothesis was partially supported with hyperactive/inattentive behaviors sharing bidirectional relations with harsh parenting tactics from age 5 to age 7. However, contrary to our hypothesis, conduct problems did not exhibit reciprocal relations with harsh parenting tactics: only conduct problems at age 3 led to increased harsh parenting at age 5. We also hypothesized that harsh parenting tactics would mediate developmental cascades from conduct problems and hyperactive/inattentive behaviors to emotional problems. This hypothesis was partially supported by the combined effect of harsh parenting and withdrawal tactics mediating a cascade from conduct problems at age 3 to increased emotional problems at age 7, thus pointing toward the value of synthesizing different developmental cascade theories. Taken together, results indicated that the reciprocal relations between harsh parenting and child behavior differ across the studied time‐lags, thus, suggesting that the relations between parenting and child behavior are influenced by developmental changes occurring during early‐ to middle‐childhood. Examining the role of (predominantly mother‐reported) withdrawal tactics in socioemotional development within an exploratory framework, we also observed developmental differences in their effect. While results suggested that withdrawal tactics may be beneficial for reducing emotional problems and hyperactive/inattentive behaviors during the preschool years, we found that they may exacerbate externalizing behaviors from age 5 to age 7. Withdrawal tactics were also seen to increase as a response to externalizing behaviors. Finally, we also explored whether there were sex differences in the relations between (predominantly mother‐reported) disciplinary parenting practices and socioemotional difficulties, finding no evidence for differences by child sex.

While a number of previous studies have found support for reciprocal relations between hyperactive/inattentive behaviors and maternal parenting behaviors (e.g., Breaux & Harvey, 2019), this is the first study to also identify these associations when using a statistical design suitable for disaggregating within‐ from between‐family effects. Conducted by Besemer et al. (2016), the only previous study to date that also investigated bidirectional relations between (primarily mother‐reported) parenting behaviors and hyperactive/inattentive behaviors using an appropriate statistical operationalization found no evidence for physical punishment, parental involvement, or parent–child communication to be associated with hyperactivity/inattention or vice versa across ages 6 to 13. Explaining their findings, Besemer et al. (2016) hypothesized that the dynamic processes implied by Patterson's coercion model may only be at play earlier in childhood, with these relations having stabilized by age 6. Investigating a slightly earlier age range, that is ages 3 to 7, findings of our study did indeed find evidence for reciprocal relations, however, this evidence was strongest between age 5 and age 7, thus only partially aligning with Besemer et al.’s hypothesis. Future studies using population representative cohorts investigating the reciprocal effects of externalizing behaviors and maladaptive parenting practices across a wider age range and including shorter time intervals between waves are needed to clarify whether such effects are indeed limited to specific developmental periods in early‐ or middle‐childhood.

From age 3 to 5, our results suggested both parent‐to‐child and child‐to‐parent effects, however, these were not reciprocal in that conduct problems but not hyperactivity/inattention were associated with subsequent increases in (predominantly mother‐reported) harsh parenting, while harsh parenting was associated with subsequent increases in hyperactivity/inattention but not in conduct problems. The absence of an effect of hyperactive/inattentive behaviors symptoms at age 3 on escalations in harsh parenting may reflect that hyperactivity is generally accepted as normative at that age (Harpin, 2005), and thus may not engender parent‐child conflict. Hyperactive/inattentive behaviors generally become increasingly salient with school entry (in the United Kingdom around age 5) when hyperactive/inattentive behaviors start to interfere with learning, but such behaviors also make it more difficult to comply with other demands that are placed on children of a certain age in the home environment (Cormier, 2008).

In contrast to hyperactive/inattentive behaviors, conduct problems were not influenced by (predominantly mother‐reported) harsh parenting tactics, suggesting that during the preschool years, such tactics do not lead to an increase in behaviors such as throwing temper tantrums. Results of the current study also refute arguments for smacking and other forms of harsh parenting which are typically based on a logic that it is necessary to use harsh parenting techniques to effectively discipline children especially when they are young and potentially do not understand verbal explanations (YouGov, 2012). In fact, our results show that harsh parenting is at best ineffective for managing conduct problems. These findings are also in line with Besemer et al.’s study (2016), adding further evidence that harsh parenting is an ineffective parenting strategy with associations of harsh parenting behaviors with increases in conduct problems mostly driven by between‐family differences. However, unlike Besemer et al. (2016), we did identify significant child‐to‐parent effects, that is, from conduct problems at age 3 on harsh parenting and withdrawal tactics at age 5. This suggests that even though aggressive behaviors do not increase as a response to (predominantly mother‐reported) harsh parenting tactics, they still lead to increased maladaptive parenting. These findings emphasize that to prevent further negative effects of such parenting behaviors on other developmental outcomes, appropriate parenting behaviors still deserve extra attention in families with children who show increased conduct problem behaviors.

Examining the effect of withdrawal tactics on externalizing and internalizing behaviors, results of the current study suggested that such tactics may have differential effects over development. Specifically, from age 3 to age 5, (predominantly mother‐reported) withdrawal tactics were associated with a reduction in hyperactive/inattentive behaviors as well as emotional problems, suggesting that during the preschool years such parenting strategies do not have a negative on effect socioemotional child development but may even reduce some unwanted behaviors. However, this effect was reversed from age 5 to age 7, indicating that withdrawal tactics may lead to increased socioemotional difficulties in older children. These differential effects are particularly interesting in the context of recent discussions on the benefit of withdrawal tactics such as “time‐outs” as these used to be a go‐to technique for managing bad behavior (Dadds & Tully, 2019). However, over the past few years, such techniques have come under criticism with some suggesting that “time‐outs” can cause children to feel rejected leading to a breakdown of secure attachment and potentially resulting in more behavioral problems (Dadds & Tully, 2019; Siegel & Bryson, 2014). Research has suggested that time‐out techniques are beneficial in reducing unwanted behaviors but should not occur too frequently and should be accompanied by explanations for why the child needs to be in time‐out, as well as by subsequent positive interactions (Dadds & Tully, 2019). One potential reason for the observed change in direction of associations could be that, at age 5, children may require more active interactions before and after a time‐out than are likely to be given by parents who frequently use withdrawal tactics. Younger children, in contrast, may still benefit from clear parenting signals indicated by withdrawal tactics even if these are not accompanied by active parenting strategies such as verbal explanations. At present, studies on the effect of withdrawal tactics are still highly limited due to the high context dependency of these behaviors. The exploratory results of the current study, however, highlight that it is important to include withdrawal tactics in discussions on reciprocal effects between children's socioemotional difficulties and disciplinary parenting tactics. Future studies looking specifically at withdrawal tactics the context they occur in, such as whether these are accompanied by active parenting strategies, are needed to disentangle the observed associations.

A further aim of the current study was to investigate the relations between (predominantly mother‐reported) harsh parenting practices and emotional problems by synthesizing Patterson's coercion model and the dual failure model. Similar to the findings on hyperactivity/inattention, we found evidence for reciprocal relations between harsh parenting and emotional problems from age 5 to age 7 but not from age 3 to age 5. Hence, these results suggest that Patterson's coercion model (2002) can also be extended to internalizing problems but potentially only for specific developmental periods. In addition, in line with the dual failure model (Capaldi, 1992) and partially in line with our hypotheses, we found that the combined effect of harsh‐parenting and withdrawal tactics mediated a developmental cascade from conduct problems at age 3 to emotional problems at age 7. This indicates that increases in harsh parenting and withdrawal tactics as a response to behavioral issues, such as aggression, may lead to later internalizing problems such as anxiety and depression, which may partially explain the high co‐occurrence rates of internalizing and externalizing problems (Kessler et al., 2005). Considering that this is the first study to observe reciprocal within‐family relations between harsh parenting and internalizing problems as well as a mediation effect of disciplinary parenting practices in cascades from conduct to emotional problems, these findings need to be replicated in other samples and should ideally also be studied over a longer period extending into and across adolescence.

Results of the current study suggested that all studied mental health domains and (predominantly mother‐reported) parenting practices showed within‐person stability (as indicated by positive autoregressive effects) across ages 3 to 5, such that, for instance, harsh parenting practices at age 3 were associated with increases in harsh parenting practices at age 5. However, this was not the case between ages 5 and 7 where only emotional problems and hyperactivity/inattention showed within‐person stability. This suggests that, for conduct problems and parenting practices, the age span of 5 to 7 represents a transition period in which parenting behaviors and conduct problems change relative to individuals’ previous behaviors. This could potentially be due to children spending more time in school, which likely leads to an adaptation in parenting strategies and to changes in conduct behaviors as children's social circles grow and peer and teacher relationships become increasingly more important.

Overall, results of the current study suggest that the reciprocal relations between parenting practices and child behavior are not stable across early‐ to middle‐childhood but that they are affected by developmental changes that occur during this period. Current developmental theories were not specific enough in guiding us to develop highly specific a‐priori hypotheses about developmental differences; thus, an important future research direction is to ensure that cascade and transactional models, such as Patterson's coercion model, more explicitly take into account developmental changes that have relevance for the transactions they address and more clearly specify any expected developmental differences.

Finally, exploration of sex differences suggested that the relations between (predominantly mother‐reported) disciplinary parenting practices and socioemotional difficulties did not differ by child sex. This suggests that while boys comparatively more often exhibit conduct problems and girls more often exhibit emotional problems (Lewis et al., 2020; Moffitt et al., 2001) preventing harsh parenting tactics is equally important for boys and girls. However, research also points to the possibility of interactions between child and parental gender. Some studies have suggested that the effect of harsh‐parenting on externalizing difficulties is stronger in same‐sex parent–child dyads (e.g. father–son) while others have suggested that it is stronger in opposite‐sex parent–child dyads (see Chang et al., 2003 for a helpful summary of the literature). Some meta‐analytic evidence has further indicated that maternal harsh parenting generally has stronger effects on children's socioemotional development than paternal harsh parenting potentially owing to the fact that mothers tend to more frequently provide daily caregiving and thus have more opportunities to influence children's socioemotional development (Rothbaum & Weisz, 1994). However, these findings are not supported by the most recent meta‐analyses on the effect of parenting behaviors on children's internalizing and externalizing difficulties (Pinquart, 2017a, 2017b). Considering that the current study predominantly relied on maternal‐reported parenting behaviors, we were not able to test for interaction effects between child sex and parental gender; thus, further research into the possibility of such differential effects in the relations between disciplinary parenting practices and socioemotional difficulties is needed.

Taken together, the current study underlines that there is a clear benefit to assessing parenting behaviors in children presenting with behavioral or emotional problems. Our findings further suggest that it would be valuable to evaluate whether interventions aiming to reduce maladaptive parenting behaviors may help interrupt the coercive cycles of parent–child interactions observed here. Interventions that teach adaptive ways of parenting, particularly focusing on positive parenting approaches that are sensitive to children's individual needs, have already shown promise for leading to a reduction in behavioral problems. For instance, the parenting intervention program “Parenting for Lifelong Health” that focus on encouraging positive parenting has been found to reduce physical and psychological discipline as well as child problem behaviors (Ward et al., 2020). Thus, such intervention strategies may help to de‐escalate parent‐child conflicts which likely leads to a reduction in socioemotional problems and can consequently help prevent negative developmental outcomes.

### Strengths and limitations

While this study had a number of strengths, including the use of a statistical operationalization that disentangles between‐ from within‐family effects, the investigation of emotional problems alongside two forms of externalizing behaviors, as well as the use of a large, population representative sample, some limitations need to be taken into account. First, both data on children's socioemotional development and on parenting tactics were primarily mother‐reported (~98%). This could have led to over‐ or underestimates of problem behaviors and use of parenting tactics and likely introduced shared‐rater bias to our models. Thus, future studies should replicate our findings using multi‐informant measures. Considering that most of the literature on the reciprocal relations between parenting practices and children's socioemotional development primarily focuses on maternal‐reported parenting, studies focusing specifically on the effect of paternal parenting behaviors are also strongly needed (e.g., Besemer et al., 2016; Breaux & Harvey, 2019). Second, considering that this study is based on a secondary data analysis of the UK representative MCS, we were limited in the time‐points available to us. Specifically, the time intervals spanned a rather large period of 2 years. Concurrent relations indicated that all domains of socioemotional development were significantly interlinked with (primarily maternal‐reported) parenting practices within the same time‐points, thus, these time‐points may not necessarily represent the ideal time‐points for investigating the reciprocal relations between parenting practices and child behavior as it is possible that these cascades might play out over shorter periods than could be observed here (Dormann & Griffin, 2015). Lastly, the measures of disciplinary parenting practices and socioemotional development were relatively short and had limited internal reliability which could have affected our results. The available parenting items, and particularly the items on withdrawal, were also somewhat vague, making it difficult to clearly define whether they indeed represent maladaptive forms of parenting. For instance, the withdrawal tactic item “ignoring” did not allow for a distinction between potentially adaptive and problematic uses of withdrawal tactics. However, the purpose of including withdrawal tactics in the current study was not to draw definitive conclusions on their effect on socioemotional development, but to provide an initial exploration of whether such parenting practices also play an important role in children's development. Thus, while the exploratory results on withdrawal tactics presented in the current study should be interpreted with caution, they clearly highlight that future studies are needed. In particular, since whether such tactics should be viewed as negative or positive likely largely depends on the context they occur in, such studies need to place parenting practices in a wider parenting context. If they are used as a form of psychological control that, for instance, manifests itself as love withdrawal, such tactics are likely to lead to later socioemotional problems. On the other hand, if withdrawal tactics such as ignoring certain unwanted behaviors occur in a context that is overall loving and nurturing and where it is clear to the child that it is not the child that is being ignored but only the behavior, such tactics can potentially be beneficial for reducing problem behaviors (Dadds & Tully, 2019; Siegel & Bryson, 2014). Thus, future studies investigating the effect of positive parenting practices such as verbal explanations alongside harsh parenting and withdrawal tactics would be highly valuable.

## CONCLUSION

To conclude, the results of our study support Patterson's coercion model as we observed reciprocal within‐family relations between (predominantly maternal‐reported) harsh parenting practices and hyperactive/inattentive behaviors as well as emotional problems. Findings not only highlight that parenting practices such as smacking, or shouting may have detrimental effects on children's mental health but also that children presenting with behavioral issues may place additional strain on maternal parenting behaviors. Consequently, it is crucial for interventions aiming to reduce the occurrence of socioemotional problems, and particularly the co‐occurrence of emotional and conduct problems, to focus on the whole family system and specifically on parenting behaviors. Furthermore, considering that harsh parenting is still used, more attention should be paid to public health campaigns that can inform parents about the potential harmful effects of such parenting practices on children's socioemotional development and equip them with alternative, more adaptive parenting tools. Finally, the findings of this study support recent changes in legislation that ban smacking in Scotland and Wales and suggest that similar legislations should also be implemented in countries such as England where smacking is still permitted as a reasonable punishment.

## CONFLICT OF INTEREST

The authors have no conflicts of interest relevant to this article to disclose.

## ETHICS STATEMENT

The MCS was approved by the London Multicentre Research Ethics Committee and is funded by the UK Economic and Social Research Council. Written consent was obtained from all participating parents at each sweep.

## Supporting information

Supplementary Material

## Data Availability

The University of London Centre for Longitudinal Studies owns the copyright for the Millennium Cohort Study (MCS) data used in this study. The MCS data are held/curated by the UK Data Service. Anyone wishing to use the MCS data (found at: https://discover.ukdataservice.ac.uk/series/?sn=2000031) must register and submit a data request to the UK Data Service at http://ukdataservice.ac.uk/. Additional terms and conditions of access are outlined here: https://www.ukdataservice.ac.uk/get‐data/how‐to‐access/conditions. Preprint: A preprint of this paper is available here: https://doi.org/10.31234/osf.io/fytw2.
