# Supplementary material for: The role of harsh parenting practices in early‐ to middle‐childhood socioemotional development: An examination in the Millennium Cohort Study
Source: Child Dev. 2022 Mar 25;93(5):1304–17. doi: 10.1111/cdev.13761 (PMC9545411; doi:10.1111/cdev.13761)
Supplement: Supplementary file 1 — Supplementary Material [file CDEV-93-1304-s001.docx]

**Supplementary Materials**

| **Table S1.** Standardised autoregressive and cross-lagged parameters | | | |
| --- | --- | --- | --- |
| **Parameter** | ***Estimate*** | ***SE*** | ***P*** |
| Age 7 emotional problems on age 5 emotional problems | 0.250 | 0.025 | **<.001*** |
| Age 7 emotional problems on age 5 hyperactivity/inattention | 0.082 | 0.021 | **<.001*** |
| Age 7 emotional problems on age 5 conduct problems | 0.090 | 0.023 | **<.001*** |
| Age 7 emotional problems on age 5 withdrawal tactics | 0.030 | 0.018 | .098 |
| Age 7 emotional problems on age 5 harsh parenting tactics | 0.036 | 0.018 | **.045*** |
| Age 7 emotional problems on age 3 hyperactivity/inattention | 0.028 | 0.023 | .216 |
| Age 7 emotional problems on age 3 conduct problems | 0.087 | 0.017 | **<.001*** |
| Age 7 hyperactivity/inattention on age 5 emotional problems | 0.024 | 0.020 | .227 |
| Age 7 hyperactivity/inattention on age 5 hyperactivity/inattention | 0.372 | 0.022 | **<.001*** |
| Age 7 hyperactivity/inattention on age 5 conduct problems | 0.061 | 0.021 | **.004*** |
| Age 7 hyperactivity/inattention on age 5 withdrawal tactics | 0.041 | 0.018 | **.020*** |
| Age 7 hyperactivity/inattention on age 5 harsh parenting tactics | 0.040 | 0.018 | **.031*** |
| Age 7 conduct problems on age 5 emotional problems | 0.118 | 0.028 | **<.001*** |
| Age 7 conduct problems on age 5 hyperactivity/inattention | 0.208 | 0.033 | **<.001*** |
| Age 7 conduct problems on age 5 conduct problems | 0.010 | 0.045 | .825 |
| Age 7 conduct problems on age 5 withdrawal tactics | 0.073 | 0.033 | **.026*** |
| Age 7 conduct problems on age 5 harsh parenting tactics | -0.026 | 0.032 | .415 |
| Age 7 withdrawal tactics on age 5 emotional problems | 0.035 | 0.026 | .174 |
| Age 7 withdrawal tactics on age 5 hyperactivity/inattention | 0.131 | 0.029 | **<.001*** |
| Age 7 withdrawal tactics on age 5 conduct problems | 0.052 | 0.040 | .190 |
| Age 7 withdrawal tactics on age 5 withdrawal tactics | 0.059 | 0.038 | .124 |
| Age 7 withdrawal tactics on age 5 harsh parenting tactics | -0.007 | 0.031 | .816 |
| Age 7 harsh parenting tactics on age 5 emotional problems | 0.086 | 0.027 | **.002*** |
| Age 7 harsh parenting tactics on age 5 hyperactivity/inattention | 0.136 | 0.032 | **<.001*** |
| Age 7 harsh parenting tactics on age 5 conduct problems | -0.034 | 0.040 | .405 |
| Age 7 harsh parenting tactics on age 5 withdrawal tactics | 0.060 | 0.030 | .050 |
| Age 7 harsh parenting tactics on age 5 harsh parenting tactics | 0.038 | 0.041 | .350 |
| Age 5 emotional problems on age 3 emotional problems | 0.087 | 0.038 | **.021*** |
| Age 5 emotional problems on age 3 hyperactivity/inattention | 0.048 | 0.030 | .108 |
| Age 5 emotional problems on age 3 conduct problems | 0.154 | 0.020 | **<.001*** |
| Age 5 emotional problems on age 3 withdrawal tactics | -0.041 | 0.020 | **.046*** |
| Age 5 emotional problems on age 3 harsh parenting tactics | 0.034 | 0.022 | .125 |
| Age 5 hyperactivity/inattention on age 3 emotional problems | 0.009 | 0.032 | .767 |
| Age 5 hyperactivity/inattention on age 3 hyperactivity/inattention | 0.150 | 0.033 | **<.001*** |
| Age 5 hyperactivity/inattention on age 3 conduct problems | 0.144 | 0.022 | **<.001*** |
| Age 5 hyperactivity/inattention on age 3 withdrawal tactics | -0.043 | 0.020 | **.029*** |
| Age 5 hyperactivity/inattention on age 3 harsh parenting tactics | 0.051 | 0.022 | **.019*** |
| Age 5 conduct problems on age 3 emotional problems | 0.017 | 0.030 | .566 |
| Age 5 conduct problems on age 3 hyperactivity/inattention | 0.029 | 0.026 | .266 |
| Age 5 conduct problems on age 3 conduct problems | 0.196 | 0.025 | **<.001*** |
| Age 5 conduct problems on age 3 withdrawal tactics | -0.030 | 0.021 | .146 |
| Age 5 conduct problems on age 3 harsh parenting tactics | -0.027 | 0.023 | .234 |
| Age 5 withdrawal tactics on age 3 emotional problems | -0.027 | 0.024 | .267 |
| Age 5 withdrawal tactics on age 3 hyperactivity/inattention | 0.013 | 0.022 | .574 |
| Age 5 withdrawal tactics on age 3 conduct problems | 0.085 | 0.022 | **<.001*** |
| Age 5 withdrawal tactics on age 3 withdrawal tactics | 0.129 | 0.023 | **<.001*** |
| Age 5 withdrawal tactics on age 3 harsh parenting tactics | 0.018 | 0.022 | .398 |
| Age 5 harsh parenting tactics on age 3 emotional problems | 0.010 | 0.025 | .701 |
| Age 5 harsh parenting tactics on age 3 hyperactivity/inattention | 0.009 | 0.024 | .694 |
| Age 5 harsh parenting tactics on age 3 conduct problems | 0.052 | 0.025 | **.035*** |
| Age 5 harsh parenting tactics on age 3 withdrawal tactics | -0.002 | 0.022 | .914 |
| Age 5 harsh parenting tactics on age 3 harsh parenting tactics | 0.152 | 0.024 | **<.001*** |
| *Note.* *Significant at p<.05. | | | |


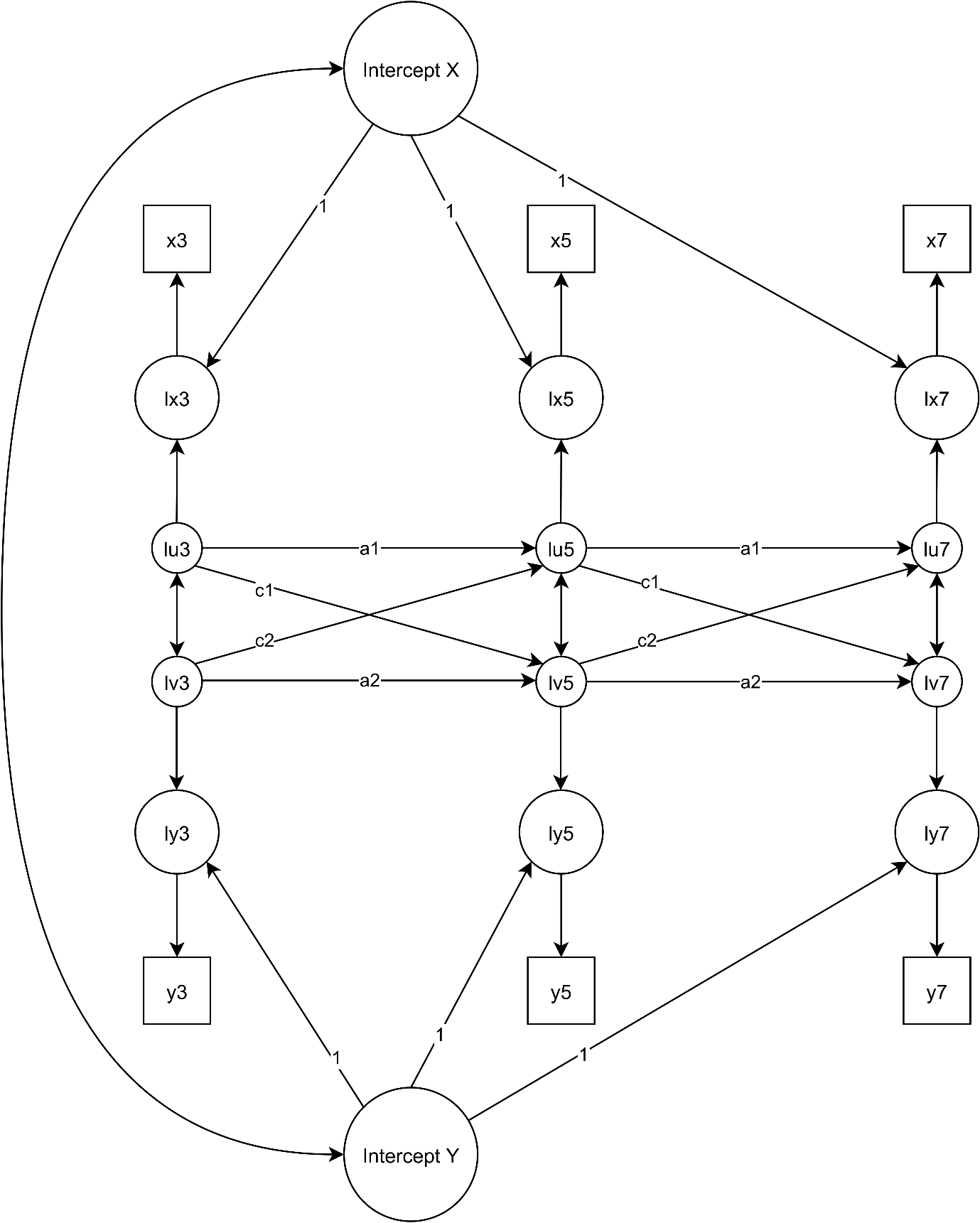


**Figure S1.** Schematic illustration of a two-outcome RI-CLPM

**Appendix 1: Failsafe *ef* calculations**

Failsafe *ef* was calculated using the following formula:

$$Standardised ef=\frac{r_{MY.X}S_{M.X}S_{Y.X}}{S_{M}S_{Y}}$$

(1)

$r_{MY.X}$ is the standardized beta coefficient for the regression of the outcome Y on the mediator M adjusting for the predictor X (Kenny, 2013). Note that in our models, this beta coefficient is also adjusted for other variables with paths to the outcome. $S_{M.X}$ and $S_{Y.X}$ are the standard deviation of the mediator adjusting for X and outcome adjusting for X respective. For ease, the standard deviations of M and Y $S_{M}$ and $S_{Y}$ were fixed to 1, making $S_{M.X}$ = $\sqrt{1-{r_{MX}}^{2}}$ and $S_{Y.X}$ = $\sqrt{1-{r_{MY.X}}^{2}}$ , where $r_{MX}$ is the standardized beta for the regression of the mediator on the predictor .

**References:**

Kenny, D. A. (2013). *Mediation: Sensitivity Analysis*. http://davidakenny.net/webinars/Mediation/Sensitivity/Sensitivity.html

**Table S2: Failsafe *ef* results**

| **Indirect effect** | $\boldsymbol{r}_{\boldsymbol{MY.X}}$ | $\boldsymbol{r}_{\boldsymbol{MX}}$ | $\boldsymbol{S}_{\boldsymbol{M}}$ | $\boldsymbol{S}_{\boldsymbol{Y}}$ | **Failsafe *ef*** | $\sqrt{\mathbf{Failsafe}\boldsymbol{ef}}$ |
| --- | --- | --- | --- | --- | --- | --- |
| Age 3 conduct problems (X) to age 7 emotional problems (Y) via age 5 harsh parenting tactics (M) | 0.036 | 0.052 | 1 | 1 | .04 | .19 |
| Age 3 conduct problems (X) to age 7 emotional problems (Y) via age 5 withdrawal tactics (M) | 0.030 | 0.085 | 1 | 1 | .03 | .17 |
| Age 3 hyperactivity/inattention (X) to age 7 emotional problems (Y) via age 5 harsh parenting tactics (M) | 0.036 | 0.009 | 1 | 1 | .04 | .19 |
| Age 3 hyperactivity/inattention (X) to age 7 emotional problems via age 5 withdrawal tactics | 0.030 | 0.013 | 1 | 1 | .03 | .17 |
